# Supplementary material for: DDT Resistance in Anopheles pharoensis from Northern Cameroon Associated with High Cuticular Hydrocarbon Production
Source: Genes (Basel). 2022 Sep 25;13(10):1723. doi: 10.3390/genes13101723 (PMC9601446; doi:10.3390/genes13101723)
Supplement: Supplementary file 1 [file genes-13-01723-s001.zip › genes-1891184-supplementary.pdf]

Supplementary Table S1. Total CHC (ng CHCs/mg mosquitos) in the populations analyzed

**mosquito Populations**	**Total CHC (ng CHCs/mg Mosquitos)**
Yangah 1	2628
Yangah 2	2121
Yangah 3	2513
Simatou 1	2587
Simatou 2	2137
Simatou 3	2391
Maga 1	1307
Maga 2	1402
Maga 3	1518

1, 2 and 3 correspond to the three biological replicates analyzed in this study
